# Co-resistance between oral antibiotics for pyelonephritis and those for cystitis—applying an escalation antibiogram model to local community data

**DOI:** 10.1093/jacamr/dlaf204

**Published:** 2025-11-10

**Authors:** Philip Williams, Edward Barton, Ranjeet Bhamber, Léo Gorman, Andrew W Dowsey, Matthew B Avison

**Affiliations:** School of Cellular and Molecular Medicine, Faculty of Life Sciences, University of Bristol, Bristol, UK; University Hospitals Bristol and Weston NHS Foundation Trust, Bristol Royal Infirmary, Bristol, UK; UK Health Security Agency, Severn Pathology, Bristol, UK; University Hospitals Bristol and Weston NHS Foundation Trust, Bristol Royal Infirmary, Bristol, UK; UK Health Security Agency, Severn Pathology, Bristol, UK; Department of Population Health Sciences, Bristol Medical School, Faculty of Health Sciences, University of Bristol, Bristol, UK; Jean Golding Institute, University of Bristol, Bristol, UK; Department of Population Health Sciences, Bristol Medical School, Faculty of Health Sciences, University of Bristol, Bristol, UK; School of Cellular and Molecular Medicine, Faculty of Life Sciences, University of Bristol, Bristol, UK

## Abstract

**Objectives:**

We applied an escalation antibiogram to community urine data to assess how presumptive resistance to first-line antibiotics for cystitis affects resistance to antibiotics used to treat pyelonephritis.

**Methods:**

We extracted susceptibility data from *Escherichia coli* isolates grown from urine samples from general practice during a 5 year period (2019–2023) in a region served by three NHS hospital trusts. Female patients over 18 years old were included, giving a total of 130 514 isolates. We applied a Bayesian model to estimate antibiotic resistance rates for oral pyelonephritis antibiotics, when presuming resistance to each of the first-line antibiotics used for cystitis. The model estimates the probability of resistance with 95% credible intervals and was applied to a variety of patient groups based on age and history of recurrent urinary tract infections.

**Results:**

Resistance to cystitis antibiotics has a marked effect on the probability of resistance to oral antibiotics used to treat pyelonephritis. In particular, amoxicillin/clavulanate should be avoided for pyelonephritis if resistance to pivmecillinam is presumed, because predicted resistance rates exceed 50%. For patients with presumed resistance to nitrofurantoin or trimethoprim, the optimal pyelonephritis antibiotic depends on both age group and history of past infections.

**Conclusions:**

Analysis using an escalation antibiogram informed by our Bayesian model is a useful tool to support empirical antibiotic prescribing for pyelonephritis. It provides an estimate of local resistance rates and a comparison of antibiotic options with a measure of the uncertainty in the data.

## Introduction

Cystitis (infection of the lower urinary tract) in women is a common presentation to primary care (in the UK, particularly general practice).^1^ A small proportion of such infections progress to infections of the upper urinary tract (pyelonephritis), which can lead to complications including renal scarring, bacteraemia and sepsis. Although evidence suggests that a significant proportion of cystitis cases would resolve without antibiotic treatment, clinical resolution is more likely when treated, and pyelonephritis is more common in cystitis not treated with antibiotics.^2^ Although not designed or powered to detect pyelonephritis, randomized controlled trials comparing cystitis antibiotic treatment with a placebo or non-steroidal anti-inflammatory drugs have demonstrated a lower rate of pyelonephritis in the antibiotic arm.^2–4^ An epidemiological study from Sweden of 752 289 women diagnosed with uncomplicated cystitis showed the case rate for pyelonephritis following cystitis was 1.61% in patients not treated with antibiotics compared with 0.55% in those treated.^5^

UK guidelines suggest first-line treatment for cystitis in adult women should be nitrofurantoin, trimethoprim, pivmecillinam or fosfomycin. Sending urine for microscopy, culture and sensitivities in women aged under 65 is recommended only for recurrent cystitis, pregnancy, or if treatment failure or pyelonephritis is suspected.^6^

If a patient initially treated for cystitis re-presents to their GP or to the emergency department with suspected pyelonephritis, they would be treated with one of the following oral antibiotics: cefalexin, ciprofloxacin, amoxicillin/clavulanate (co-amoxiclav) and, if in hospital, gentamicin would also be an option.^7^ Ciprofloxacin has been the mainstay of pyelonephritis treatment due to low resistance rates compared with other oral options and proven efficacy.^8^

Prescribing decisions are complex, and the antibiotic choice with the lowest resistance rate may not be preferred due to other considerations, such as side effects,^9,10^ pregnancy^11,12^ and renal function.

Although in some cases treatment can be guided by recent urine susceptibility results, in many cases pyelonephritis therapy would be empirical. Over a 4 year period from 2020 to 2023 our local emergency department at the Bristol Royal Infirmary treated 1769 cases of pyelonephritis. Positive microbiology (from blood culture or a midstream urine sample) was only available in 30% of cases. This anecdotal account is supported by a published cohort study of 105 pyelonephritis cases from 2015, which found that 50% had positive microbiology.^13^

Between 75% and 90% of pyelonephritis is caused by *Escherichia coli*, and antibiotic guidelines for pyelonephritis are usually based on *E. coli* sensitivities.^14^ A number of studies have demonstrated that resistance to one antibiotic is associated with increased probability of resistance to other antibiotics, and three recent studies have investigated aspects of co-resistance in *E. coli* from urine samples. Guy *et al.*^15^ explored the use of nitrofurantoin resistance as a marker for MDR in samples from the UK. Kaye *et al.*^16^ described co-resistance between four antibiotic classes (nitrofurantoin, co-trimoxazole, fluoroquinolones and third-generation cephalosporins) in the USA. Leroy *et al.*^17^ investigated co-resistance amongst oral antibiotics recommended for pyelonephritis in France, concluding that an oral option is almost always available.

Treatment for cystitis has a reported failure rate of between 4% and 16%. Risk factors for empirical treatment failure in cystitis include increased age, prior antibiotic use and comorbidities, each of which are also risk factors for increased antimicrobial resistance (AMR).^18–21^ One study found higher failure rates in antibiotics with higher local resistance rates,^20^ though UK data^18^ suggest repeat prescriptions with the same antibiotic occur in 20% of treatment failures, indicating resistance is not always suspected. Although the failure of initial cystitis treatment does not guarantee resistance to the initial antibiotic, the possibility of resistance should be considered when prescribing follow-up treatment.

The term ‘escalation antibiogram’ has been coined to describe an antibiogram generated to guide empirical therapy assuming resistance to other antibiotics.^22^ Accordingly, using local *E. coli* susceptibility data from general practice urine samples, we applied a Bayesian model initially developed to guide empirical antibiotic escalations in suspected Gram-negative sepsis^23^ to predict the resistance rate to antibiotics used locally for pyelonephritis treatment. We did this for scenarios where resistance to any one of our first-line cystitis antibiotics was assumed and where widespread susceptibility testing is performed (nitrofurantoin, trimethoprim and pivmecillinam).

## Methods

### Data collection

Data were collected from a region served by three NHS trusts covering four acute hospitals in the southwest of England (Royal United Hospital Bath NHS Foundation Trust, University Hospitals Bristol & Weston NHS Foundation Trust, and North Bristol NHS Trust), which share a single laboratory information management system (Winpath Enterprise 7.23, Clinisys). All *E. coli* grown from urine samples sent from GPs over a 5 year period, 2019 to 2023 inclusive, in this region were included. Resistance data were extracted for antibiotics used for cystitis (nitrofurantoin, trimethoprim and pivmecillinam) and pyelonephritis (co-amoxiclav, cefalexin, ciprofloxacin and gentamicin). Repeat isolates in the same patient within 28 days were removed as representing one episode of infection. For each isolate, the antibiotic susceptibility profile was determined using EUCAST disc testing methodologies (v8.0 to v12.0). Results were expressed as ‘susceptible’ or ‘resistant’, with intermediate results classed as resistant. We extracted these susceptibility data for female patients over 18 years old, giving a total of 130 514 isolates. Recurrent urinary tract infection (UTI) was defined as three or more urine samples in a 12 month period. Data from 2018 were used to identify this patient subgroup in the 2019 data. The distribution of tested urine samples by age is included in the Supplementary material (available as Supplementary data at JAC-AMR Online).

### Bayesian model

We used a Bayesian spline model with a Bernoulli likelihood and logit link function to model antimicrobial susceptibility test resistance rates over time, fitted by Hamiltonian Monto Carlo sampling. The model generates a series of ‘credible’ curves to fit the resistance data, each with the same probability of representing the true rate given the inherent uncertainty. To avoid overfitting, an integrated penalization term adaptively smooths the curves given the level of evidence. Statistics such as mean and credible intervals can be directly computed from samples of the curves at the required times. More details are given in a previous article.^23^ Resistance rates between two groups or timepoints were compared by subtracting one set of posterior predictive curves from another, allowing us to calculate the posterior probability of an increase (PPI) or decrease (PPD) in resistance over time, or to calculate posterior probability of inferiority (PPInf) or superiority (PPSup) between two antibiotic options.

Although the model is applicable to any patient group, in this article we have used data from female patients, as this group has the highest burden of cystitis and pyelonephritis. Predicted antibiotic resistance rates for pyelonephritis antibiotics with 95% credible intervals were calculated for the end of December 2023 based on the previous 5 years of data for each patient subgroup.

### Subgroup selection

Pyelonephritis is most common in women aged between 18 and 29 years, and rates increase again from the age of 50 onwards.^24^ For women over 65 years, cystitis becomes increasingly common, with a rate of infection twice that of adult women as a whole.^25^ This is associated with higher rates of resistance and, in addition to this, urine samples are routinely sent in this age group, rather than being sent only for complex or recurrent infections. To reflect these differences in epidemiology and sample collection we considered three age groups in our research: 18–50, 51–65 and over 65 years. In addition to looking at all urine samples for each age group, we also looked at the first sample sent for each patient in a 12 month period (i.e. no prior samples) and at patients with recurrent UTI, defined as three or more urine samples being sent within a 12 month period (i.e. two or more prior samples). Note that the first sample sent for each patient in the under-65 age group may not represent their first infection, so our data may overestimate the resistance expected for a patient’s actual first presentation.

## Results

For female patients aged over 18 years within our region, resistance rates for *E. coli*, from GP-submitted urine samples for ciprofloxacin, cefalexin, co-amoxiclav and gentamicin were sufficiently low that any of these three antibiotics could be used empirically to treat pyelonephritis (see Table 1).

**Table 1.** Posterior mean resistance estimates (95% credible intervals) for urinary *E. coli* for pyelonephritis antibiotics: all female patients from GP samples

Age group	UTI samples	Number	Cefalexin	Ciprofloxacin	Co-amoxiclav	Gentamicin
18–50	All	30 572	0.08 (0.07–0.09)	0.06 (0.05–0.07)	0.08 (0.07–0.10)	0.04 (0.03–0.05)
18–50	First	23 590	0.07 (0.06–0.08)	0.05 (0.04–0.06)	0.08 (0.07–0.09)	0.04 (0.03–0.05)
18–50	Two plus	2552	0.12 (0.08–0.16)	0.10 (0.07–0.14)	0.09 (0.06–0.13)	0.07 (0.03–0.10)
18–50	Pregnant	2557	0.09 (0.05–0.13)	0.04 (0.07–0.12)	0.11 (0.07–0.17)	0.04 (0.02–0.07)
51–65	All	22 994	0.07 (0.06–0.08)	0.06 (0.05–0.07)	0.09 (0.07–0.10)	0.05 (0.04–0.06)
51–65	First	15 084	0.07 (0.05–0.08)	0.05 (0.04–0.06)	0.08 (0.10–0.07)	0.05 (0.04–0.06)
51–65	Two plus	3647	0.09 (0.06–0.15)	0.09 (0.06–0.12)	0.11 (0.08–0.14)	0.08 (0.05–0.12)
Over 65	All	66 567	0.07 (0.06–0.08)	0.07 (0.06–0.08)	0.09 (0.08–0.10)	0.05 (0.05–0.06)
Over 65	First	34 455	0.06 (0.05–0.07)	0.06 (0.05–0.07)	0.07 (0.06–0.09)	0.04 (0.03–0.05)
Over 65	Two plus	17 288	0.09 (0.08–0.11)	0.09 (0.08–0.10)	0.12 (0.10–0.13)	0.07 (0.06–0.09)

Using our escalation antibiogram approach^22^ we investigated how resistance to first-line cystitis antibiotics (nitrofurantoin, trimethoprim and pivmecillinam) among urinary *E. coli* alters the expected resistance rates of pyelonephritis antibiotics for all patients and in a range of clinically important subgroups. Our methodology can be applied to any subgroup of clinical interest that can be identified within the available pathology metadata. The smaller the group of interest, the greater the uncertainty in the results, which is reflected in the wider 95% credible intervals, but the data may more accurately reflect the microbiology of that group and its antibiotic exposures.

We investigated how the optimal pyelonephritis treatment options vary with age group and the number of positive urine samples sent per patient in the previous 12 months, which we use as a proxy for prior antibiotic exposure.

**Figure 1.**
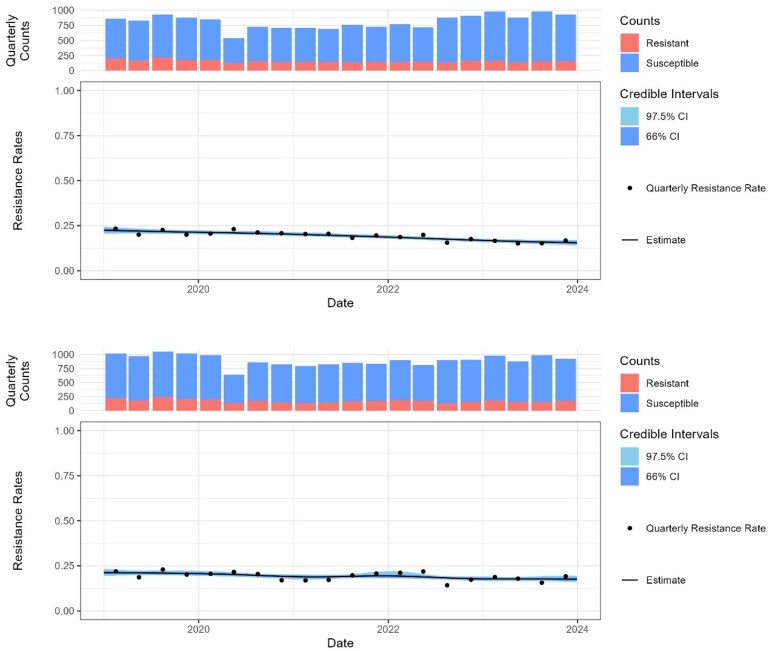
Percent resistance to ciprofloxacin (top) and co-amoxiclav (bottom) assuming trimethoprim resistance, in all samples from women over 65 years of age. The black line shows the posterior probability of resistance, and the shading the 66% and 97.5% credible intervals.

The results for all samples and the selected subgroups are given in Tables 1–4. When comparing antibiotic options for a large dataset, a small difference in resistance rate can be reproducible but not clinically significant. For example, in *E. coli*–positive urine samples from women over 65 (*n* = 66 567), where there is resistance to trimethoprim, the model estimates that 15.8% (95% CI, 14.1–17.6) will be co-resistant to ciprofloxacin and 17.0% (95% CI, 14.2–19.4) co-resistant to co-amoxiclav. We can calculate it is 90% likely that resistance to ciprofloxacin is lower in this trimethoprim-resistant population, but this difference is of little clinical significance (Figure 1 and Table 4).

**Table 2.** Posterior mean resistance estimates (95% credible intervals) for urinary *E. coli* for pyelonephritis antibiotics, given resistance to pivmecillinam, varying by age and number of UTIs in the previous 12 months

Age group	UTI samples	Number	Cefalexin	Ciprofloxacin	Co-amoxiclav	Gentamicin
18–50	All	30 572	0.09 (0.05–0.17)	0.06 (0.02–0.11)	0.59 (0.48–0.72)	0.12 (0.07–0.18)
18–50	First	23 590	0.09 (0.04–0.19)	0.04 (0.01–0.09)	0.60 (0.48–0.72)	0.11 (0.06–0.17)
18–50	Two plus	2552	0.17 (0.04–0.42)	0.15 (0.01–0.39)	0.50 (0.26–0.77)	0.23 (0.06–0.48)
19–50	Pregnant	2557	0.10 (0.01–0.29)	0.05 (0.00–0.19)	0.62 (0.37–0.86)	0.16 (0.02–0.38)
51–65	All	22 994	0.09 (0.05–0.15)	0.08 (0.04–0.13)	0.57 (0.48–0.66)	0.12 (0.07–0.18)
51–65	First	15 084	0.08 (0.03–0.14)	0.08 (0.03–0.14)	0.53 (0.41–0.64)	0.11 (0.05–0.19)
51–65	Two plus	3647	0.09 (0.02–0.23)	0.08 (0.01–0.20)	0.48 (0.32–0.66)	0.16 (0.04–0.40)
Over 65	All	66 567	0.10 (0.07–0.13)	0.14 (0.10–0.17)	0.51 (0.42–0.59)	0.12 (0.09–0.17)
Over 65	First	34 455	0.09 (0.05–0.14)	0.12 (0.08–0.18)	0.49 (0.39–0.57)	0.11 (0.07–0.16)
Over 65	Two plus	17 288	0.13 (0.09–0.19)	0.15 (0.09–0.22)	0.59 (0.48–0.70)	0.15 (0.24–0.09)

**Table 3.** Posterior mean resistance estimates (95% credible intervals) for urinary *E. coli* for pyelonephritis antibiotics, given resistance to nitrofurantoin, varying by age and number of UTIs in the previous 12 months

Age group	UTI samples	Number	Cefalexin	Ciprofloxacin	Co-amoxiclav	Gentamicin
18–50	All	30 572	0.20 (0.09–0.36)	0.35 (0.20–0.52)	0.16 (0.05–0.31)	0.14 (0.04–0.29)
18–50	First	23 590	0.26 (0.09–0.54)	0.26 (0.07–0.53)	0.24 (0.07–0.56)	0.05 (0.00–0.20)
18–50	Two plus	2552	0.21 (0.03–0.45)	0.43 (0.10–0.75)	0.11 (0.01–0.33)	0.33 (0.08–0.63)
18–50	Pregnant	2557	0.49 (0.10–0.84)	0.67 (0.25–0.98)	0.47 (0.14–0.85)	0.80 (0.39–0.99)
51–65	All	22 994	0.16 (0.06–0.28)	0.08 (0.00–0.21)	0.19 (0.08–0.33)	0.06 (0.02–0.17)
51–65	First	15 084	0.28 (0.11–0.53)	0.15 (0.02–0.39)	0.12 (0.01–0.34)	0.05 (0.00–0.18)
51–65	Two plus	3647	0.09 (0.01–0.27)	0.07 (0.00–0.39)	0.29 (0.09–0.68)	0.15 (0.01–0.64)
Over 65	All	66 567	0.29 (0.20–0.40)	0.17 (0.11–0.25)	0.23 (0.16–0.33)	0.14 (0.08–0.25)
Over 65	First	34 455	0.11 (0.03–0.21)	0.10 (0.04–0.18)	0.16 (0.07–0.28)	0.13 (0.05–0.26)
Over 65	Two plus	17 288	0.39 (0.26–0.55)	0.23 (0.13–0.37)	0.28 (0.17–0.42)	0.12 (0.05–0.22)

**Table 4.** Posterior mean resistance estimates (95% credible intervals) for urinary *E. coli* for pyelonephritis antibiotics, given resistance to trimethoprim, varying by age and number of UTIs in the previous 12 months

Age group	UTI samples	Number	Cefalexin	Ciprofloxacin	Co-amoxiclav	Gentamicin
18–50	All	30 537	0.16 (0.19–0.13)	0.12 (0.11–0.13)	0.16 (0.13–0.20)	0.15 (0.10–0.16)
18–50	First	23 590	0.16 (0.13–0.20)	0.12 (0.09–0.14)	0.15 (0.12–0.19)	0.13 (0.10–0.16)
18–50	Two plus	2553	0.20 (0.13–0.30)	0.19 (0.12–0.28)	0.16 (0.10–0.24)	0.16 (0.09–0.23)
18–50	Pregnant	2499	0.20 (0.12–0.32)	0.15 (0.06–0.29)	0.19 (0.10–0.31)	0.12 (0.04–0.22)
51–65	All	22 994	0.14 (0.11–0.18)	0.12 (0.09–0.14)	0.17 (0.14–0.20)	0.15 (0.12–0.18)
51–65	First	15 084	0.14 (0.11–0.17)	0.11 (0.08–0.13)	0.15 (0.11–0.20)	0.14 (0.11–0.17)
51–65	Two plus	3647	0.17 (0.10–0.27)	0.16 (0.10–0.23)	0.20 (0.14–0.28)	0.17 (0.11–0.25)
Over 65	All	66 567	0.12 (0.11–0.14)	0.16 (0.14–0.18)	0.17 (0.14–0.19)	0.14 (0.12–0.16)
Over 65	First	34 455	0.11 (0.08–0.13)	0.12 (0.10–0.15)	0.15 (0.12–0.18)	0.10 (0.07–0.13)
Over 65	Two plus	17 288	0.15 (0.12–0.19)	0.20 (0.17–0.23)	0.21 (0.18–0.24)	0.20 (0.15–0.26)

**Figure 2.**
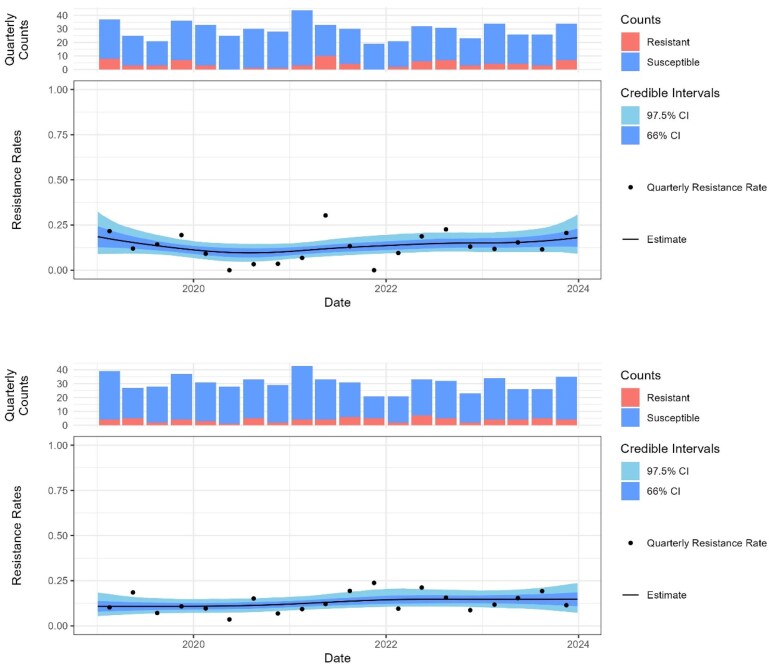
Percent resistance to ciprofloxacin (top) and co-amoxiclav (bottom) assuming trimethoprim resistance, in pregnant women. The black line shows the posterior probability of resistance, and the shading the 66% and 97.5% credible intervals.

For small patient groups, where there is greater uncertainty in the data, a direct comparison between two antibiotic options may demonstrate similar probability of success despite an apparent difference in the posterior probability of resistance. For example, in *E. coli*–positive urine samples from pregnant women (*n* = 2499) with resistance to trimethoprim, 15.4% (95% CI, 6.4–28.7) are predicted to be co-resistant to ciprofloxacin and 20.5% (95% CI, 11.7–32.5) to cefalexin. In this case despite a difference of 5% points in predicted resistance rates, the credible intervals overlap to the extent that there is a probability of 23.7% that resistance is actually lower for cefalexin (Figure 2).

## Discussion

We now consider the consequence of resistance to each of the three cystitis antibiotics in turn, assessed using our Bayesian approach, on predicted resistance to pyelonephritis antibiotics. These analyses are informed by microbiology data at population level. Their application to the treatment of individual patients would be guided by likely resistance to a first-line cystitis antibiotic, indicated by treatment failure, or by the observation of resistance in a recent urine sample.

### Pivmecillinam

**Figure 3.**
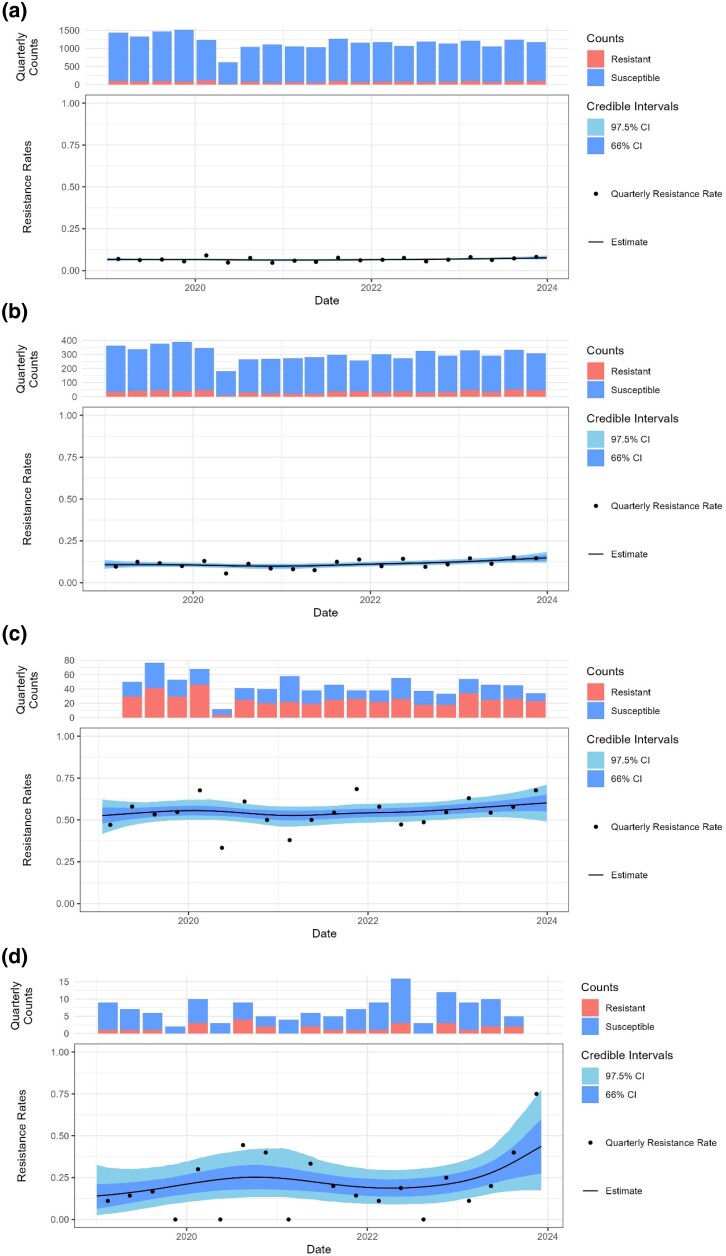
Predicted resistance to co-amoxiclav in women aged 18–50 years with no prior UTIs in the previous 12 months. (a) Overall resistance, (b) assuming resistance to trimethoprim, (c) assuming resistance to pivmecillinam and (d) assuming resistance to nitrofurantoin. The black line shows the posterior probability of resistance, and the shading the 66% and 97.5% credible intervals.

In patients where pivmecillinam resistance is likely, co-amoxiclav should be avoided as expected resistance rates are between 50% and 60% (Table 2). Hyper-production of TEM-1 β-lactamase in *E. coli* has been reported to confer resistance to both co-amoxiclav and pivmecillinam,^26^ so this may explain the high rate of co-resistance observed here (Figure 3).

The expected resistance rates to ciprofloxacin and cefalexin were acceptable in all patient groups when resistance to pivmecillinam was present. Assuming no contraindications, cefalexin would generally be preferred given its favourable side effects profile. The highest rates of expected resistance were in patients aged 18–50 years with recurrent UTIs. Here cefalexin resistance was 16.7% (95% CI, 3.5–42) and ciprofloxacin resistance was 15.1% (95% CI, 1.2–39). This difference is unlikely to be clinically significant (Table 2).

Given resistance to pivmecillinam, we found the expected resistance rate in pregnant women was 10% (95% CI, 1.3–29) for cefalexin compared with 5.4% (95% CI, 0.4–18.6) for ciprofloxacin. Cefalexin would still be preferred in this patient group (Table 2).

**Figure 4.**
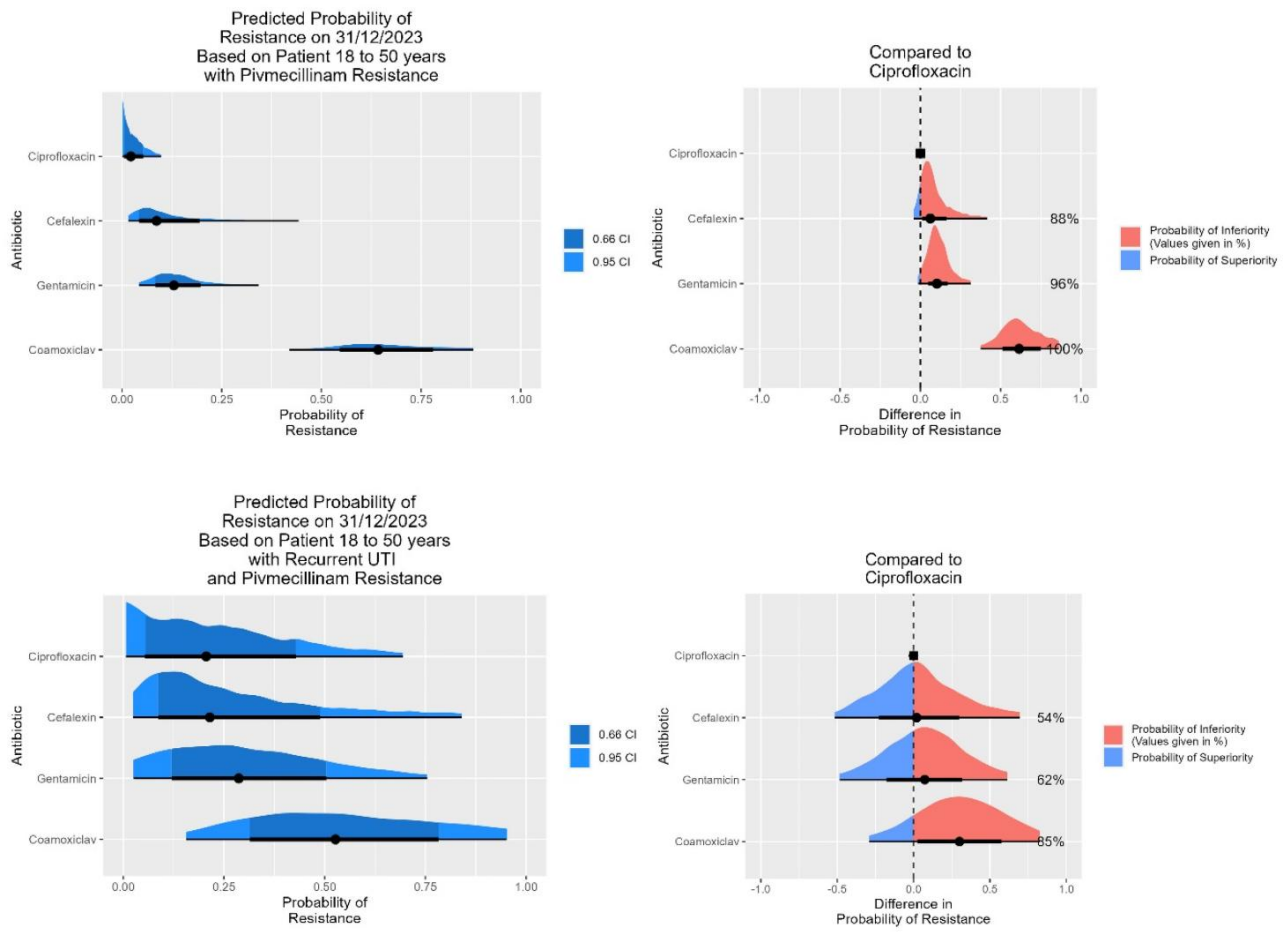
Predicted resistance to four pyelonephritis antibiotics assuming resistance to pivmecillinam with credible intervals, with each antibiotic compared with ciprofloxacin. Top: female patients in the 18–50 age group with no prior UTIs, and (bottom) with recurrent UTIs.

Figure 4 shows how the model can be used graphically to display the probability of resistance to the four pyelonephritis antibiotics being considered here, and to calculate the probability of greater resistance to three of these compared with that with the lowest rate of resistance (ciprofloxacin). This example looks at women aged 18–50 years assuming pivmecillinam resistance, and with no prior samples nor with recurrent UTIs. We can see that for those with no prior UTIs, ciprofloxacin has the lowest expected resistance rate, but that cefalexin is an acceptable option, whereas for those with recurrent UTIs, ciprofloxacin and cefalexin have a similar but higher rate of expected resistance.

### Nitrofurantoin

Nitrofurantoin resistance is rare in urinary *E. coli* from women aged under 50 in our data, with an overall resistance rate of 1% (95% CI, 0.5–1.5), rising to 5.5% (95% CI, 3.2–8.6) in those with recurrent UTIs. This small resistant population results in higher uncertainty (thus wider 95% CI) in expected resistance to pyelonephritis antibiotics (Figure 3).

Resistance rates in this age group were expected to be lowest for co-amoxiclav when resistance to nitrofurantoin was present. For patients with no prior samples all three oral options (co-amoxiclav, cefalexin and ciprofloxacin) have a similar expected resistance rate (Table 3).

In pregnant women where nitrofurantoin resistance was suspected, all pyelonephritis treatment options (including IV gentamicin) had a high rate of expected resistance with a wide 95% CI. For example, cefalexin resistance rate was estimated to be 49% but with a 95% CI of 10–84. Although cefalexin is most likely to be the best option (lower risk in pregnancy), the high resistance rate and high level of uncertainty suggest careful monitoring of the patient’s clinical status, and a low threshold for hospital admission would be appropriate. Note that nitrofurantoin should be avoided near term but may be used at other stages of pregnancy.

Given resistance to nitrofurantoin in the older patient groups, our data suggest co-amoxiclav and ciprofloxacin would be favoured over cefalexin (Table 3).

### Trimethoprim

In our region, trimethoprim resistance was common in urinary *E. coli*, varying from 26.4% (95% CI, 24–28.7) in women aged under 50 years with no prior samples, to 38% (95% CI, 33.5–42.9) for women aged over 65 years with recurrent UTIs. This larger population positive for resistance means a narrow 95% CI for expected resistance to pyelonephritis treatments (Figure 3).

Expected resistance rates were lower for ciprofloxacin than for cefalexin or co-amoxiclav for patients aged under 65 years where trimethoprim resistance is likely. Due to the potential side effects of ciprofloxacin, co-amoxiclav or cefalexin are likely to be favoured when resistance rates are low enough for these agents to be viable options.

Cefalexin would be favoured in pregnancy despite a higher expected resistance rate of 20.5% (95% CI, 11–32) than ciprofloxacin at 15.4% (95% CI, 6.4–28.7), due to a high level of uncertainty (there is a 23.7% probability that resistance to cefalexin is actually lower; see Figure 2) and possible risk of adverse fetal outcomes. Trimethoprim is contraindicated in the first trimester but can be used later in pregnancy.

For patients over 50 years old with trimethoprim-resistant organisms, cefalexin had lower expected resistance rates than ciprofloxacin, with co-amoxiclav having the highest rates of resistance (Table 4).

### Limitations of the study

This study used local pathology data to provide insights into the local AMR with the aim of improving empirical prescribing. This sampling itself will be a source of bias, for which we have no method of correcting.

The data can be subdivided to provide data more closely matched to a particular patient’s risk factors for resistance. We can, however, only subdivide the populations based on metadata recorded in the pathology record. We have investigated subgroups based on age and previous urine samples sent. Although the methodology could be applied to any subgroup that can be identified, we do not currently have data on comorbidities or direct data on prior antibiotic exposures.

Our laboratory made no changes to the breakpoints applied to urine samples during the study period. Data that include MIC values (including automated systems such as Vitek or Phoenix) allow the modification of the dataset following a change in breakpoints.^23^ Data based on disc testing cannot be adjusted following a change in breakpoints. Following the implementation of a change in breakpoints, there can be an apparent jump (or fall) in the rate of resistance. A period of data collection post change would be required for the model to stabilize.

We have focused on antibiotics currently recommended in our national guidelines.^7^ The IDSA guidelines for the treatment of complicated UTI raise a concern that therapy with oral β-lactam agents may be inferior to other regimens.^27^ There are conflicting data published on the comparative efficacy of oral β-lactams to either trimethoprim/sulfamethoxazole or fluoroquinolones for the treatment of serious infections caused by Gram-negative organisms, with some studies demonstrating inferiority and others showing equivalence or non-inferiority. A meta-analysis did not demonstrate any increase in mortality when β-lactams were used as step-down therapy for Enterobacterales bloodstream infection, although there was an increased risk of recurrence, which may reflect suboptimal dosing.^28^

### Conclusions

To the best of our knowledge, this is the first example of the escalation antibiogram being applied to community data from the UK and could lead to improved antibiotic prescribing for pyelonephritis, which is an important condition.

Antibiotic resistance and co-resistance patterns will differ from region to region and country to country, hence the results of this study are unlikely to be directly transferable. The methodology, however, is simple to apply where the underlying data are routinely collected. If antibiotic resistance among urinary pathogens increases over time, we are likely to see more pyelonephritis due to initial treatment failure for cystitis and to see higher resistance rates to second-line agents. This will increase the utility of our approach to optimize the escalation of antibiotic treatment. We recognize that antibiotic selection decisions are complex, balancing the likely resistance rates against side effect profiles, drug–drug interactions and other patient-specific factors, so these estimated resistance rates will feed into that process, rather than provide a definitive answer to the prescriber.

Within our region we have demonstrated clinically significant differences in rates of expected resistance to pyelonephritis antibiotics when resistance to first-line cystitis antibiotics is accounted for. We suggest this model could be applied to local data in other regions by infection specialists and then simplified to generate practical guidelines for GPs, emergency departments and acute medical units.

Within our region we would suggest:

- In pregnancy, cefalexin would be first choice and gentamicin second in all cases.
- With suspected or confirmed resistance to pivmecillinam, in any age group, then treat with cefalexin in the first instance and ciprofloxacin second. Avoid the use of co-amoxiclav.
- With suspected or confirmed resistance to trimethoprim, treatment with cefalexin is optimal in patients aged over 65. In patients under 65, ciprofloxacin has the lowest resistance rate, but either cefalexin or co-amoxiclav may be used due to similar rates of resistance and preferred side effect profiles.
- With suspected or confirmed resistance to nitrofurantoin then use co-amoxiclav first line, with, cefalexin second line. Ciprofloxacin would be a third option.

## Supplementary Material

dlaf204_Supplementary_Data
