# Supplementary figures and images for: Co-resistance between oral antibiotics for pyelonephritis and those for cystitis—applying an escalation antibiogram model to local community data
Source: JAC Antimicrob Resist. 2025 Nov 10;7(6):dlaf204. doi: 10.1093/jacamr/dlaf204 (PMC12599318; doi:10.1093/jacamr/dlaf204)

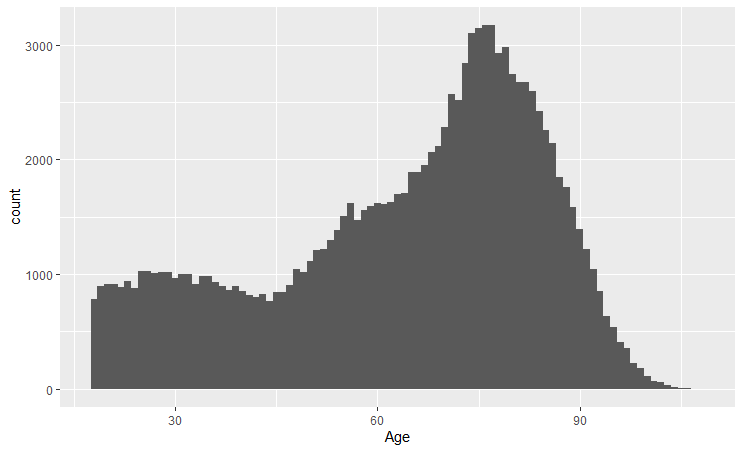


Distribution of urine samples by age


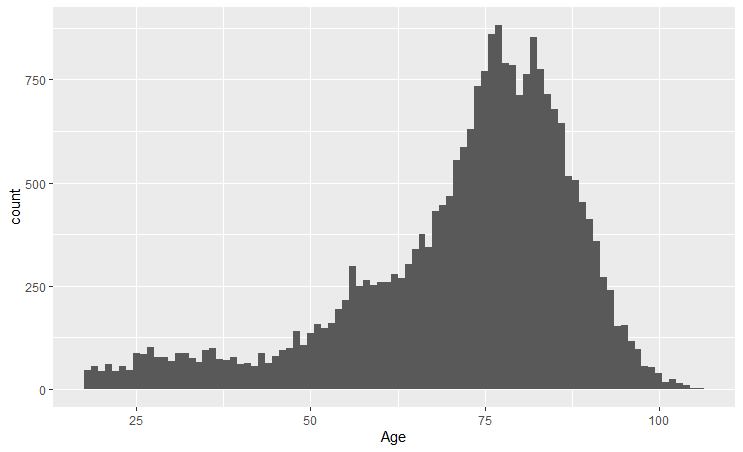


Distribution of Samples by age for recurrent UTIs

Supplement: dlaf204_Supplementary_Data [file dlaf204_supplementary_data.docx]
